# A quantitative model for spatio-temporal dynamics of root gravitropism

**DOI:** 10.1093/jxb/erad383

**Published:** 2023-10-23

**Authors:** Amir Porat, Mathieu Rivière, Yasmine Meroz

**Affiliations:** School of Physics and Astronomy, Tel Aviv University, Tel Aviv 6997801, Israel; School of Plant Sciences and Food Security, Tel Aviv University, Tel Aviv 6997801, Israel; School of Plant Sciences and Food Security, Tel Aviv University, Tel Aviv 6997801, Israel; INRAE-LEPSE, Montpellier, France

**Keywords:** Curvature, dynamics, experiment, gravitropism, mathematical model, root, spatiotemporal, tropism

## Abstract

Plant organs adapt their morphology according to environmental signals through growth-driven processes called tropisms. While much effort has been directed towards the development of mathematical models describing the tropic dynamics of aerial organs, these cannot provide a good description of roots due to intrinsic physiological differences. Here we present a mathematical model informed by gravitropic experiments on *Arabidopsis thaliana* roots, assuming a subapical growth profile and apical sensing. The model quantitatively recovers the full spatio-temporal dynamics observed in experiments. An analytical solution of the model enables us to evaluate the gravitropic and proprioceptive sensitivities of roots, while also allowing us to corroborate the requirement for proprioception in describing root dynamics. Lastly, we find that the dynamics are analogous to a damped harmonic oscillator, providing intuition regarding the source of the observed oscillatory behavior and the importance of proprioception for efficient gravitropic control. In all, the model provides not only a quantitative description of root tropic dynamics, but also a mathematical framework for the future investigation of roots in complex media.

## Teaser

We develop a mathematical model for spatio-temporal dynamics of root gravitropism informed by experiments, identifying the dynamic contributions of proprioception and passive orientation drift.

## Introduction

Plants are sessile organisms, physically anchored to the ground. They survive and prosper in fluctuating environments by adapting their morphology via tropisms—the redirection of growth according to external stimuli, such as light and gravity (Gilroy and Masson, 2007; Muthert *et al.*, 2020; Meroz, 2021). Broadly speaking, gravitropism is triggered when statocytes sense the inclination of the organ relative to the direction of gravity, based on the sedimentation of statoliths (Hashiguchi *et al.*, 2013; Chauvet *et al.*, 2016; Pouliquen *et al.*, 2017), and the growth hormone auxin is redistributed, resulting in differential cell elongation causing the organ to bend.

**Fig. 1.**
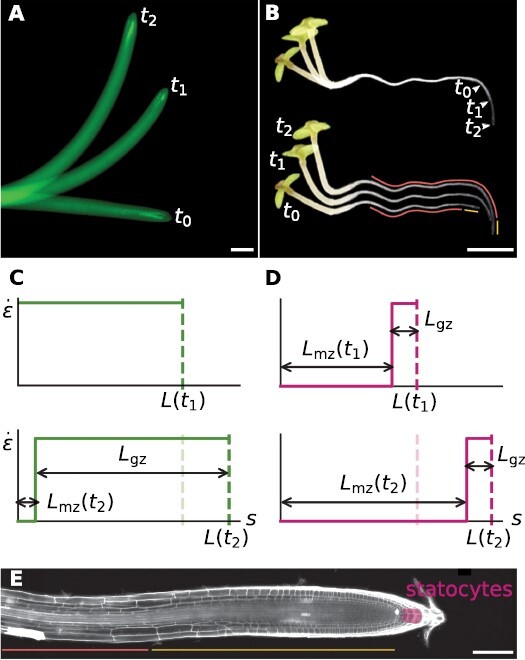
Gravitropism in roots and shoots. (A) Negative gravitropic response of a coleoptile of wheat (*Avena sativa*), with snapshots at *t*_0_ = 0, *t*_1_ = 4, and *t*_2_ = 8 h. (B) Gravitropic response of an *Arabidopsis thaliana* seedling with snapshots at *t*_0_ = 0, *t*_1_ = 7, and *t*_2_ = 14 h. The hypocotyl (left) displays negative gravitropism, while the root (right) shows positive gravitropism. The overlaid picture of the same root at different times (top) demonstrates how the trajectory of the tip matches the final shape of the root. The bottom image, where root images are shown separately, shows how the curvature is produced in the growth zone (yellow line) and progressively fixed in the mature zone (red line). Scale bar = 2 mm. (C) Schematic representation of the spatial distribution of the elongation rate along a growing wheat coleoptile at the beginning of gravistimulation (top), and at the end of the experiment (bottom). We mark the length of the whole root *L*(*t*), the mature zone *L*_mz_(*t*), and the growth zone *L*_gz_(*t*)_._ (D) Idem for the Arabidopsis root. (E) Micrograph of an *A. thaliana* root. The columella (pink patch) is an order of magnitude smaller than the growth zone (yellow line) and contains inclination-sensitive cells called statocytes that are at the foundation of gravisensing. Scale bar = 100 μm (Courtesy of Eilon Shani).

The fundamental role of tropisms in the adaptive capabilities of plants has motivated a number of coarse-grained mathematical models describing their macroscopic dynamics (Guillon *et al.*, 2012; Bastien *et al.*, 2013, 2014; Bressan et al., 2017; Agostinelli *et al.*, 2020; Moulton *et al.*, 2020; Porat *et al.*, 2020). While such models are macroscopic in nature, their conclusions can guide the investigation of biological principles underpinning tropisms, providing testable hypotheses relating macroscopic dynamics to microscopic processes, for example statolith sedimentation and the redistribution of auxin (Pouliquen *et al.*, 2017; Moulton *et al.*, 2020; Levernier *et al.*, 2021). Furthermore, articulating biological theories using mathematical language, informed by experimental data, has the advantage (Autran *et al.*, 2021) of providing (i) a ‘compressed’ understanding of large amounts of experimental data with different parameters and conditions and (ii) quantitative predictions based on mathematical analyses, facilitating hypotheses testing. As an example, analysis of the mathematical description of shoot gravitropism led to the identification of the requirement for proprioception (sensing of local curvature) for the regulation of the gravitropic response (Bastien *et al.*, 2013). While roots are a favorable model for the investigation of genetic and biochemical mechanisms underlying gravitropism (Binenbaum *et al.*, 2018; Fendrych *et al.*, 2018; Kaiser *et al.*, 2023) due to their advantages in imaging and microscopy, macroscopic models of tropisms have generally been tailored for shoots. However, there are significant differences between shoot and root gravitropism, reflected by different spatio-temporal dynamics. As an example, Fig. 1A and B shows snapshots during the gravitropic responses of wheat coleoptiles (turning away from gravity) and *Arabidopsis thaliana* roots (growing towards the direction of gravity). While almost the whole coleoptile changes shape in time, in roots most of the shape changes occur close to the tip becoming frozen in time, and the final shape of the root closely matches the trajectory of the tip (Chavarría-Krauser *et al.*, 2008). These differences are generally attributed to physiological differences such as the extent of the growth zone where growth occurs and curvature is produced, and the distribution of the sensory system (Bastien *et al.*, 2014, 2015). A schematic plot of the size and position of the growth zone relative to the organ length during the gravitropic responses shown in Fig. 1A and B, is presented in Fig. 1C and D, respectively. Most roots grow by several times the length of their growth zone, and therefore most of the produced curvature is frozen in the mature zone (where growth no longer occurs). Conversely shoots grow by only a fraction of length, and changes in shape occur along most of the coleoptile. As for gravisensing, in aerial organs this is local, with statocytes distributed along the whole epidermis in dicots (Hawker, 1932; Fujihira *et al.*, 2000), or along the vascular bundle in monocots (Hawker, 1932; Sack *et al.*, 1983; Kutschera, 2001). In roots, gravisensing is apical, with statocytes confined to the columella (Blancaflor *et al.*, 1998) located in the root cap. The relative size of the columnella, which is an order of magnitude smaller than the growth zone in an *Arabidopsis* root, is illustrated in Fig. 1E.

These different dynamics call for an immediate need for a mathematical description of root gravitropic dynamics. Here we conduct gravistimulation experiments on *A. thaliana* roots, and quantitatively characterize their dynamics. This characterization informs the development of a mathematical model, building on the ‘angle–curvature–elongation’ (ACE) model (Bastien *et al.*, 2014) together with the said differences between root and shoot gravitropism, which accurately describe the observed spatio-temporal dynamics of gravitropic responses of *Arabidopsis* roots.

## Materials and methods

### Plant material and growth conditions

The wild-type strain of *A. thaliana* was used in this study. Sterile seeds were plated on a growth medium consisting of half-strength Murashige and Skoog medium adjusted to pH 5.7, 1% sucrose, and 1% phytagel. Seeds were stratified for 2 d at 4 °C and then kept vertical in a growth chamber at 24 °C under a 12 h photoperiod.

### Experimental protocol

After germination, seedlings were transferred to another Petri dish under a biological hood. Seedlings were arranged by groups of five and the roots were placed between the lid of the Petri dish and a block of fresh phytagel (same content as the growth medium). This new Petri dish was then mounted on a rotating plate. During the experiment, the plate was maintained vertical for 10 h, then tilted horizontally for the following 24 h. White light was provided from the top throughout the experiment, except when taking pictures (light from all directions). Pictures were taken every 10 min using a Nikon D750 camera. The inclination angle, lighting, and camera were all controlled by an Arduino micro-controller running a specifically developed program.

### Image analysis

**Fig. 2.**
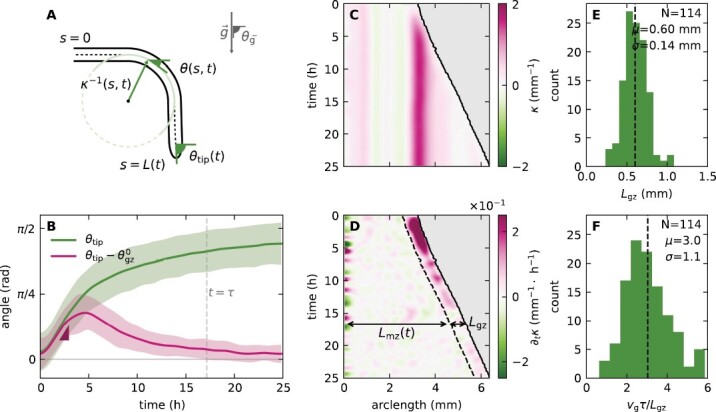
Characterization of tropic responses of Arabidopsis roots. (A) Geometrical description. θ(*s*,*t*) is the local angle of the root at point *s* along the organ, at time *t*. The local curvature κ(*s*,*t*) = ∂θ(*s*,*t*)/∂s represents how strongly the local angle changes along the organ. *s* = 0 at the base and *s* = *L*(*t*) at the tip, where *L*(*t*) is the organ length, and *r* is the radius. θ_tip_ is the angle at the tip, and θ_g_ = π/2 is the direction of the gravity signal. (B) Average evolution of the tip angle (green) and difference between the angles at the tip and base of the growth zone (pink) during the gravitropic response of *A. thaliana* roots tilted horizontally. The vertical dashed line indicates τ, the typical time needed for θ_tip_ to reach a steady state. (C) Graphical representation (kymograph) of the evolution of κ(*s*,*t*) along a specific root. (D) Kymograph of ∂κ(*s*,*t*)/∂*t*, highlighting that changes in curvature occur within the growth zone, allowing assessment of *L*_gz_ from the observed dynamics. (E) Distribution of measured values of *L*_gz_, with an average value of *L*_gz_ = 0.6 mm. (F) Distribution of the total growth of roots over their gravitropic response, *L*(τ)–*L*_0_ = *v*_g_τ, in units of *L*_gz_, where *v*_g_ is the apical growth velocity. On average, Arabidopsis roots grow three times the *L*_gz_ during their gravitropic response, meaning that a mathematical description of the dynamics has to take an explicit account of growth.

The noise and contrast of the acquired pictures were edited using the free software RawTherapee (http://rawtherapee.com/) to ease further analysis steps. We used a version of Interekt (Hartmann *et al.*, 2022) to extract the shape of each root at each time step of the experiment, yielding the root arc length *s*, its local angle with respect to the horizontal θ(*s*,*t*), the total length *L*(*t*), and the radius *r*(*s*,*t*) (see Fig. 1F). The radius was averaged over both *s* and *t*. Assuming linear growth, the tip growth velocity *v*_g_ = *v(s* = *L*(*t*),*t*) was extracted as the slope of a linear fit of *L*(*t*). We defined three further angles: (i) the tip angle θ_tip_ = θ(*s* = *L*(*t*),*t*) and (ii) the angle at the base of the growth zone, at the interface of the mature zone, $\theta_{gz}^{0}=\theta \left(s=L\left(t\right)-L_{gz},t\right)$. Both were obtained by locally averaging θ(*s*,*t*) over a characteristic length of 0.1 mm around the points of interest. Lastly, (iii) the tip angle in the final steady state θ_f_, defined as the average of θ_tip_(*t*) over the last 5 h of the experiment, and σ(θ_f_) the corresponding standard deviation. The curvature κ(*s*,*t*) was calculated according to κ = ∂θ/∂*s*. We define the time to reach gravitropic equilibrium τ as the time for θ_tip_(*t*) to approach its steady state value θ_f_ ± σ(θ_f_). Assuming that growth is the only driver of curvature changes as in the ACE model, we estimated the growth zone *L*_gz_ indirectly by looking at the extent over which curvature changes over time ∂κ(*s*,*t*)/∂*t*≠0, as clearly seen in Fig. 2D. We estimate *L*_gz_ from $\dot{\kappa }\left(s\right)$ the spatial profile of ∂κ/∂*t* in the frame attached to the tip and averaged in time for *t* < τ. *L*_gz_ is then defined as the minimal distance from the tip such that $\dot{\kappa }\left(s\right)>0.05{\langle \dot{\kappa }\rangle }_{s}$, where ${\langle \cdot \rangle }_{s}$ denotes averaging over *s* (Supplementary Fig. S1). The growth rate in the growth zone is approximated according to ${\dot{\varepsilon }}_{0}\approx v_{g}/L_{gz}$.

### Curve fitting

The fitting of experimental trajectories of θ_tip_(*t*) to Equation 9 and the second-order ordinary differential equation (ODE) in Equation 8 was done using the curve_fit function from the open source SciPy Python library. This function called the odeint function from the same library, which solved the ODE multiple times using the free fitting parameters {γ,η,θ_0_} until the fitting was complete. Lastly, since both $\tilde{\theta }=\theta_{0}-\theta_{g}$ and $\tilde{\theta }=-\theta_{0}-\theta_{g}$ yield the same initial condition for θ_g_ = π/2, we bounded the fitting of θ_0_ using θ_0_ > 0.

## Results and discussion

### Characterization of growth in the gravitropic response of Arabidopsis roots

We start with a quantitative characterization of growth as a driver of the tropic response, evaluating the extent of the growth zone relative to the region over which tropic bending occurs. The shape of a root of length *L*(*t*) at time *t* is described by θ(*s*,*t*), the local angle at point *s* along the centerline, where *s* = 0 at the base and *s* = *L*(*t*) at the tip (Fig. 2A). The local curvature is defined as the rate at which the angle changes along the centerline, κ(*s*,*t*) = ∂θ(*s*,*t*)/∂*s*. The growth zone extends from the tip along a length *L*_gz_. We define θ_tip_(*t*) = θ(*s* = *L*(*t*),*t*) the angle at the tip, and $\theta_{gz}^{0}=\theta \left(s=L\left(t\right)-L_{gz},t\right)$ the angle at the base of the growth zone.

These variables provide insight into the dynamics of the gravitropic response (an extended list of the variables used here appears in Supplementary Table S1). Figure 2B shows the evolution of the root tip angle θ_tip_(*t*) over time, during permanent gravistimulation experiments. Upon being tilted horizontally, the tip angle starts at an angle 0, and increases until it approximately realigns with the direction of gravity. It approaches its steady-state value θ_f_ ± σ(θ_f_) over a time scale τ (see the Materials and methods), with the whole growth zone approaching a flat steady state shape, with $\theta_{tip}-\theta_{gz}^{0}\approx 0$.

A full description of the spatio-temporal dynamics is given by the curvature κ(*s*,*t*). A typical example is presented in Fig. 2C, showing that κ(*s*,*t*) increases within the growth zone near the apex after gravistimulation, and goes back to 0 after a few hours, as manifested in $\theta_{tip}-\theta_{gz}^{0}$. The curvature developed in the growth zone becomes fixed in the mature zone, where growth no longer occurs. This behavior is highlighted further in ∂κ(*s*,*t*)/∂*t* (Fig. 2D), where we can identify the growth zone as the region near the tip where changes in curvature occur with ∂κ(*s*,*t*)/∂*t*≠0. Outside of the growth zone ∂κ(*s*,*t*)/∂*t* ≈ 0, and the curvature remains constant (see also Supplementary Fig. S1). This analysis, described in the Materials and methods for multiple roots, enables us to estimate *L*_gz_ for each root and yields an average value of *L*_gz_ = 0.60 ± 0.14 mm (Fig. 2E).

For each root we compare *L*_gz_ with the length grown during the whole gravitropic response (detailed in the Materials and methods). We find that by the time roots reach their final angle at *t* = τ, they grow on average three times the growth zone, namely *v*_g_τ = (3.0 ± 1.1)*L*_gz_ (Fig. 2F), where *v*_g_ is the tip growth velocity. This result, in addition to the curvature fixation in the mature zone, confirms that the effects of growth cannot be neglected, as can be done in some cases (Bastien *et al.*, 2013).

### The root model

Based on the variables defined before, we build on the ACE model (Bastien *et al.*, 2013, 2014) for tropic dynamics with an explicit account of growth. This model has been analyzed and corroborated experimentally for aerial organs (Bastien *et al.*, 2013, 2014), and has recently been generalized to 3D (Agostinelli *et al.*, 2020; Moulton *et al.*, 2020; Porat *et al.*, 2020). The organ length *L*(*t*) evolves according to an axial growth rate profile $\dot{\varepsilon \;}\left(s,t\right)$, such that the local growth velocity is $v\left(s,t\right)=\overset{s}{\underset{0}{\int }}\dot{\varepsilon }\left(u,t\right)du$. Within the growth zone $\dot{\varepsilon \;}\left(s,t\right)\neq 0$, and the general form of the ACE model reads


$$\begin{array}{l} \frac{r}{\dot{\varepsilon }(s,t)}\frac{D\kappa (s,t)}{Dt}=-\beta \sin\left(\theta (s,t)-\theta_{g}\right)-\gamma r\kappa (s,t) \end{array}$$ (1)

where *r* is the radius of the organ, θ_g_ is the direction of the stimulus (here the direction of gravity), β is the gravitropic sensitivity or gain, γ is the proprioceptive sensitivity, and *D*/*Dt* = ∂/∂*t* + *v*(*s*,*t*) ∂/∂*s* is the material derivative due to growth. The organ is clamped at the base such that θ(*s* = 0,*t*) = θ_0_ and, in the mature zone *D*κ(*s*,*t*)/*Dt* = 0.

In general, we distinguish between two phases of growth during organ development: *exponential* growth refers to the initial stage where the growth zone spans the length of the whole organ [the case for coleoptiles as illustrated in Fig. 1C, taking $\dot{\varepsilon \;}\left(s,t\right)={\dot{\varepsilon }}_{0}$ for simplicity], which increases exponentially over time. *Linear* growth refers to the next phase when some tissues stop elongating and *L*(*t*) > *L*_gz_. Only the subapical part of the organ elongates (as for roots, illustrated in Fig. 1D and E), and the total length of the organ increases linearly with time.

As discussed before, in order to describe the dynamics of root growth, one must implement apical sensing and linear growth. Equation 1 can be modified for apical sensing by replacing θ(*s*,*t*) with θ_tip_(*t*), and assuming a constant growth rate $\dot{\varepsilon }\left(s,t\right)={\dot{\varepsilon }}_{0}$ for simplicity, reading in the growth zone


$$\begin{array}{l} \frac{r}{{\dot{\varepsilon }}_{0}}\frac{D\kappa (s,t)}{Dt}=-\beta \sin\left(\theta_{tip}(t)-\theta_{g}\right)-\gamma r\kappa (s,t) \end{array}$$ (2)

which we will term here the *root model*.

While the steady-state shape has been described analytically in the simpler case of exponential growth and local sensing (Bastien *et al.*, 2014), the investigation of linear growth has been limited to numerical studies. Here we focus on a detailed analysis of the root model.

We note that here we do not take into account the elastic properties of roots and assume that their shape is not altered by external forces. We base our assumption on the fact that the roots grow on vertical substrates with minimal resistance, and self-weight effects are negligible due to their low mass, as discussed in previous studies (Bastien *et al.*, 2013, 2015; Chelakkot and Mahadevan, 2017).

### Solution of the root model

In order to solve Equation 2, we express the tip angle θ_tip_ as a function of curvature, recalling that by definition $\theta_{tip}\left(t\right)-\theta_{0}=\overset{L(t)}{\underset{0}{\int }}\kappa \left(s,t\right)ds$. We simplify this relationship by distinguishing between the curvature in the growth zone (GZ) and that in the mature zone (MZ), and splitting the integral accordingly.

In the case of apical sensing the signal from the apex is uniform along the growth zone, and assuming an initially straight organ the resulting curvature is also uniform along the growth zone. We therefore rewrite the curvature in the growth zone as a function of time alone κ(*s*,*t*) = κ_gz_(*t*). In the mature zone, curvature is fixed, and is therefore a function of arc-length alone, so κ(*s*,*t*) = κ_mz_(*s*). While *L*_gz_ is fixed over time, the mature zone increases. Since the growth velocity is the integral of the growth rate along the growth zone, the organ length increases linearly according to $L\left(t\right)=L_{0}+L_{gz}{\dot{\varepsilon }}_{0}t$, where *L*_0_ is the initial length of the organ such that *L*_0_ > *L*_gz_. The length of the mature zone is then


$$\begin{array}{l} L_{mz}(t)=L(t)-L_{gz}=L_{0}+L_{gz}\;{\dot{\varepsilon }}_{0}t-L_{gz} \end{array}$$ (3)

We now simplify the expression for θ_tip_(*t*) by splitting the integral over the growth zone and mature zone, and substituting κ_gz_(*t*) and κ_mz_(*s*) accordingly:


$$\begin{array}{lll} \theta_{tip}\;\left(t\right)-\theta_{0}\; & =\int_{0}^{L_{mz}\left(t\right)}\kappa_{mz}\left(s\right)ds+\int_{L_{mz}\left(t\right)}^{L\left(t\right)}\kappa_{gz}\left(t\right)ds\; & =\int_{0}^{L_{mz}\left(t\right)}\kappa_{mz}\left(s\right)ds+L_{gz}\kappa_{gz}\left(t\right) \end{array}$$ (4)

To continue, we note that the curvature at a point *s* in the mature zone κ_mz_(*s*) was fixed when it exited the growth zone at some previous time *t*ʹ < *t*, which allows it to be related to a past value of the curvature of the growth zone κ_mz_(*s*) = κ_gz_(*t*ʹ). Since the basal end of the growth zone is equivalent to the length of the mature zone, the time *t*ʹ at which arc length *s* left the growth zone can be found by *s* = *L*_mz_(*t*ʹ). This means that κ(*s*,*t*) of the entire organ is always continuous but not smooth at *s* = *L*_mz_(*t*). Assuming for simplicity that *L*_0_ = *L*_gz_, Equation 3 yields $s=L_{mz}\left(t^{\prime }\right)={\dot{\varepsilon }}_{0}L_{gz}t^{\prime }$, which allows us to change variables in the last integral in Equation 4 from *s* to *t*ʹ:


$$\begin{array}{l} \theta_{tip}\left(t\right)-\theta_{0}={\dot{\varepsilon }}_{0}L_{gz}\int_{0}^{t}\kappa_{gz}\left(t^{\prime }\right)dt^{\prime }+L_{gz}\kappa_{gz}\left(t\right) \end{array}$$ (5)

We substitute Equation 5 into Equation 2. The material derivative now becomes a simple time derivative since κ_gz_(*t*) is as a function of time alone. Together, the partial differential equation of the original ACE model is now transformed to an ordinary integro-differential equation for κ_gz_(*t*):


$$\begin{array}{l} \frac{r}{{\dot{\varepsilon }}_{0}}\frac{d\kappa_{gz}}{dt}=-\beta sin\left({\dot{\varepsilon }}_{0}L_{gz}\int_{0}^{t}\kappa_{gz}\left(t^{\prime }\right)dt^{\prime }+L_{gz}\kappa_{gz}+{\tilde{\theta }}_{0}\right)-\gamma r\kappa_{gz} \end{array}$$ (6)

where ${\tilde{\theta }}_{0}=\theta_{0}-\theta_{g}$ is the initial angle relative to the gravitational stimulus.

We rewrite Equation 6 in a non-dimensional form: (i) normalizing curvature according to *L*_gz_, defining *k* = *L*_gz_κ_gz_ equivalent to the angle traced by the growth zone $\theta_{tip}-\theta_{gz}^{0}$; and (ii) normalizing time according to the growth rate, defining $\tau ={\dot{\varepsilon }}_{0}t=L_{mz}\left(t\right)/L_{gz}$ equivalent to the organ growth in units of *L*_gz_. Lastly, we define η = β*L*_gz_/*r* (experimental values listed in Table 1), where *L*_gz_/*r* is the geometric slenderness ratio of the growth zone, and η acts as an effective gravitropic sensitivity. Substituting these non-dimensional quantities in Equation 6, and denoting d*k*/dτ ≡ *k*ʹ, yields

**Table 1.** Measured slenderness ratios and root model (RM) fitted variables with *R*^2^ > 0.9 (88 out of 114 roots).

Definition	Notation	Average value
Root radius	*r*	65 ± 11 μm
Growth zone length	*L*_gz_	0.60 ± 0.14 mm
Slenderness ratio	*L*_gz_/*r*	9.3 ± 2.3
Tip growth velocity	*v*_g_	0.13 ± 0.04 mm h^–1^
Effective gravitropic sensitivity	η (RM)	1.1 ± 0.6
Gravitropic sensitivity	β (RM)	0.12 ± 0.06
Gravitropic sensitivity	β (bend)	0.14 ± 0.06
Proprioceptive sensitivity	γ (RM)	1.6 ± 1.0
Oscillation’s quality factor	*Q* (RM)	0.44 ± 0.16
Critical damping	γ_crit_ (RM)	0.9 ± 0.1


$$-\frac{1}{\eta }\left(k^{'}+\gamma k\right)=\sin\left({\int_{0}^{\tau }k\left(\tau^{'}\right)d\tau^{'}+k+\tilde{\theta }}_{0}\right)$$ (7)

Applying arcsin and taking another derivative in τ results in


$$\begin{array}{l} -\frac{(k^{\prime \prime }+\;\gamma k^{\prime })}{\sqrt{\eta^{2}-{\left(k^{\prime }+\gamma k\right)}^{2}}}=k+k^{\prime } \end{array}$$ (8)

Equation 8 is a second-order non-linear ODE for *k*(τ) with the initial conditions *k*(0) = 0 and $k^{\prime }\left(0\right)=-\eta \;sin({\tilde{\theta }}_{0})$. We note that a straight organ, with *k*(τ) = 0, is a steady-state solution. This equation can be easily solved numerically, and we find the evolution of θ_tip_(*t*) by substituting the solution of Equation 8$k(\tau )=k({\dot{\varepsilon }}_{0}t)$ in the normalized form of Equation 5:


$$\begin{array}{l} \theta_{tip}\left(t\right)=\theta_{0}+\int_{0}^{{\dot{\varepsilon }}_{0}t}\;\;k(\tau^{\prime })d\tau^{\prime }+k\left({\dot{\varepsilon }}_{0}t\right) \end{array}$$ (9)

### Qualitative analysis of root model solutions and comparison with other models

**Fig. 3.**
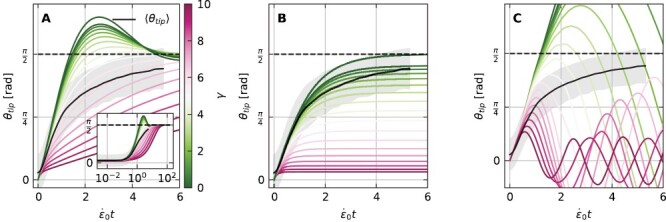
Simulations of gravitropic responses of Arabidopsis roots for different models. (A) Root model. Simulated evolution of θ_tip_ solving the root model, obtained by numerically solving Equation 8, and substituting the solution into Equation 9. Here θ_g_ = π/2, θ_0_ = 0, and η = βL_gz_/*r* = 1 for proprioceptive values γ ranging from 0 to 10. Over long times, the tip angle always reaches the direction of gravity θ_g_ = π/2 marked by a dashed line, as also found in experiments in Fig. 2, here plotted by a black line. (B) AC model. Evolution of θ_tip_ for the AC model (Bastien *et al.*, 2013), where growth is implicitly assumed to be the driver of the tropic response, but not taken into account explicitly. Simulations use the same parameters as in (A). The tip aligns with the stimulus only in the case of γ = 0. (C) ACE model with exponential growth. Evolution of θ_tip_ for the ACE model (Bastien *et al.*, 2014), where the growth zone is assumed to include the whole organ. Simulations use the same parameters as in (A) and (B). The tip overshoots the direction of the stimulus in all cases.

The evolution of θ_tip_(*t*) solved for the gravitropic response of a root tilted horizontally, with θ_0_ = 0, θ_g_ = π/2, η = β*L*_gz_/*r* = 1 [based on typical values for *Arabidopsis* found in the literature (Roué *et al.*, 2019), see Table 1], and for a range of proprioception values γ is shown in Fig. 3A. In all cases the tip eventually aligns with the direction of gravity, and the averaged experimental trajectory follows a similar trend. We later quantitatively discuss the fit of experimental trajectories with root model solutions. For comparison, Fig. 3B and C show the corresponding simulated trajectories for the AC model, which does not account for growth, and the ACE model with exponential growth, both with apical sensing. In the Supplementary data we develop the analytical solutions plotted here for both models. In the AC model, the tip angle never aligns with the direction of gravity apart from the case with no proprioception γ = 0, and by definition it does not account for growth, describing only cases with $L\left(t\right)/L_{gz}\ll 1$. We also recall that in the case of apical sensing, the AC model leads to a constant curvature throughout the root organ, which, as we discuss later, does not reflect the spatial growth pattern of roots. Despite these inconsistencies, the AC model manages to fit the tip angle trajectories well, and in the Supplementary data we compare its fitting results with those of the root model (Supplementary Fig. S2). Lastly, the trajectories of the ACE model with exponential growth exhibit significant oscillations, overshooting the direction of gravity and never reaching a steady state. The deviation from experimental trajectories is such that attempts to fit to the exponential ACE model do not converge.

### Fitting tip angle trajectories and parameter estimation

We fit θ_tip_(*t*) trajectories of the gravitropic experiments to numerical solutions of the root model, Equations 8 and 9, detailed in the Materials and methods. The fitting parameters are the proprioceptive sensitivity γ, the effective gravitropic sensitivity η = β*L*_gz_/*r*, and the initial angle θ_0_. The fit therefore provides an estimation of these parameters, and 77% of experiments fit with *R*^2^ > 0.9 (Supplementary Fig. S2). The rest of the parameters, *r*, *v*_g_, and *L*_gz_, are extracted directly through image analysis, while ${\dot{\varepsilon }}_{0}$ is estimated *viav*_g_/*L*_gz_ [in Supplementary Fig. S3 we show that a varying ${\dot{\varepsilon }}_{0}$ profile, e.g. triangular (Quiros *et al.*, 2022), has a negligible effect]. Parameters are summarized in Table 1.

**Fig. 4.**
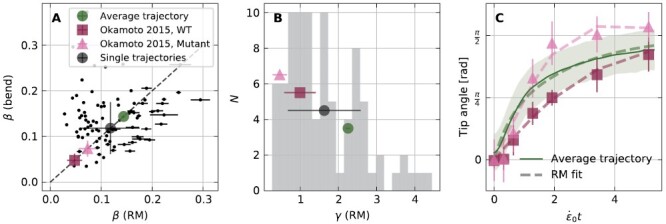
Estimation of gravitropic and proprioceptive sensitivities β and γ by fitting tip angle trajectories to the root model. (A) The gravitropic sensitivity β estimated using two methods: (i) fitting the θ_tip_ trajectories measured for single Arabidopsis roots with solutions of the root model from Equation 9 (with *R*^2^ > 0.9, see Supplementary Fig. S2), which gives β = 0.12 ± 0.06 and (ii) extracted by the maximal initial bending (Equation 10) which gives β = 0.14 ± 0.06. Single trajectories are plotted (black dots) along with their ensemble statistics (large black circle). (B) Histogram (shaded area) of the proprioceptive coefficient values γ estimated by fitting θ_tip_ trajectories to solutions of the root model (with *R*^2^ > 0.9, see Supplementary Fig. S2). The average value is γ = 1.6 ± 1.0 (black circle). (C) Fit of averaged trajectories using the root model solutions for three different datasets: (i) the solid green line represents the average trajectory of our data and the dashed line represents the fitted root model solution with β = 0.14 ± 0.00 and γ = 2.3 ± 0.1; (ii) purple squares represent average trajectories for the wild type from Okamoto *et al.* (2015), and the dashed line represents the root model fit with β = 0.05 ± 0.01 and γ = 1.0 ± 0.4; and (iii) pink triangles represents hyper-bending mutants from Okamoto *et al.* (2015), and the dashed line represents the root model fit with β = 0.07 ± 0.02 and γ = 0.5 ± 0.2. Colored symbols representing the fitted β and γ also appear in (A) and (B).


We compare the values of β (Fig. 4A) estimated for single trajectories using the root model with an alternative estimation method based on the maximal bending of the growth zone (Chauvet *et al.*, 2016):

$$\begin{array}{l} \beta_{bend}\approx \max\frac{d}{dt}\left(\theta_{tip}(t)-\theta_{gz}^{0}(t)\right)r/v_{g} \end{array}$$ (10)

where *v*_g_ is the tip growth velocity (slope shown in Fig. 2B).

The two methods agree on the average gravitropic sensitivity, with average values $\langle \beta_{RM}\rangle =0.12\pm 0.06$ and $\langle \beta_{bend}\rangle =0.14\pm 0.06$, respectively.

**Fig. 5.**
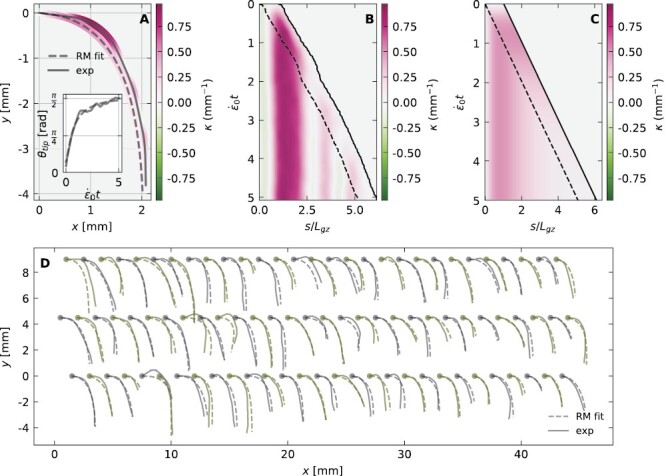
Root model recovers full spatio-temporal dynamics of organ shape. (A) Example of a specific Arabidopsis root shape (solid line) overlaid with the simulated shape using the parameters extracted from a fit to the θ_tip_ trajectory using the solutions of the root model. The color represents curvature, as in the kymographs in (B) and (C). The inset shows the corresponding θ_tip_ trajectory (solid black) along with its fit (dashed line) based on Equation 9 with a coefficient of determination *R*^2^ = 0.98. The fitted sensitivities are γ = 1.6 ± 0.0 and β = 0.17 ± 0.00. (B) Graphical representation (kymograph) of the evolution of κ(*s*,*t*) along a specific root, whose tip angle and shape are presented in (A). The constant growth zone *L*_gz_ and increasing mature zone *L*_mz_ are delineated with a dashed line. (C) The simulated kymograph based on the root model fit. An animated version of (A–C), comparing the full spatio-temporal shape dynamics of the experiment and the root model, appears in Supplementary Video S2 (see the Supplementry data for details). (D) Estimated shapes given by the root model against their corresponding experimental measured centerlines, 24 h after the roots were tilted perpendicular to gravity. The estimation of the root shape was done using the parameters given by the fit of the root model to their tip angle trajectory, and the roots appearing here were fitted with *R*^2^ > 0.9. A quantification of the error in these shape estimation appears in Supplementary Fig. S6. Note that the error in the shape is larger for roots with a negative θ_0_ (tilted upwards at the base) as it is bound to positive values in our fitting scheme (see the Materials and methods).

This further corroborates the root model, and we now proceed to evaluate the proprioceptive coefficient γ—the first systematic estimation for roots. The distribution of the values of γ estimated by fitting the root model for single θ_tip_ trajectories, with an average value 〈
γ〉
=1.6±1.0 is presented in Fig. 4B. We attempted to fit the trajectories to a modified root model with γ = 0. Only 5% of trajectories fit well with *R*^2^ > 0.9 (Supplementary Fig. S2), confirming that proprioception is required in the description of root growth dynamics. To emphasize the difference in the resulting shape dynamics, we animate the gravitropic turns given by the root model solutions with and without proprioception in Supplementary Video S1 (see the Supplementary data for details). Furthermore, we analyzed data from Okamoto *et al.* (2015) for ACTIN8, an *Arabidopsis* mutant defective in myosin XI exhibiting hyper-bending in its gravitropic response, and predicted to correspond to a lower proprioceptive gain. We fit the averaged θ_tip_ trajectories for 35 wild-type roots and 35 mutant roots digitized from Okamoto *et al.* (2015), as well as the average trajectory from Fig. 2, using the root model (Fig. 4C). Estimated values of β and γ are summarized in Table 2 and Fig. 5 (colored symbols), where as predicted the proprioceptive value for the overshooting mutant is lower than for the wild type. As values for *v*_g_, *r*, and *L*_gz_ were not provided, and we used our values instead (Table 1), the direct comparison between values extracted from Okamoto *et al.* (2015) and our own data is limited.

**Table 2.** Estimations of β and γ based on the root model using data from Okamoto *et al.* (2015).

	β	γ
Wild type	0.05 ± 0.01	1.0 ± 0.4
Mutant	0.07 ± 0.02	0.5 ± 0.2

The hyper-bending mutants were fitted with a lower proprioceptive coefficient than the wild type.

### The root model recovers full spatio-temporal dynamics of the organ

The tip angle dynamics provides limited insight into the shape of the whole organ throughout the response. For example, while the AC model with apical sensing provides a good description of θ_tip_ trajectories (Supplementary Fig. S2), it wrongly predicts a uniform curvature throughout the organ (Fig. 2C). We analyze and compare the full spatio-temporal dynamics of roots and the root model. An example of the final shape of a specific root is shown in Fig. 5A, where colors represent the local curvature κ(*s*). The dashed line represents the simulated root, based on the parameters estimated from the fit to θ_tip_ of the same root (shown in the inset), in agreement with the observed form. A kymograph of the measured κ(*s*,*t*) of the root over time is presented in Fig. 5B, and Fig. 5C shows the simulated κ(*s*,*t*) of the same root. In order to compare experiment with simulation, we normalize time with ${\dot{\varepsilon }}_{0}t$ and space with *s*/*L*_gz_. The growth zone *L*_gz_ is marked on both figure panels with a dashed line. We find that the simulation captures the main features of the spatio-temporal dynamics, namely the initial production of curvature in the growth zone over short times, which is then fixed at the base of the organ (Chavarría-Krauser *et al.*, 2008). We note that despite the similarity with the simulated root, the curvature in the growth zone of actual roots is not uniform, as illustrated and quantified in Supplementary Figs S4 and S5.

 The final experimental root shapes compared against the predicted shapes are shown in Fig. 5D for cases where the tip angle trajectories fit with *R*^2^ > 0.9. Visual inspection suggests a good agreement. We quantify the difference between the experimental and predicted shapes (Supplementary Fig. S6), according to two scores: (i) ${\langle |{\vec{x}}_{exp}-{\vec{x}}_{RM}|\rangle }_{s}/r=2.54\pm 1.53$, the average distance between points along the centerlines, normalized by the measured radius of the root; and (ii) ${\langle |\theta_{exp}-\theta_{RM}|\rangle }_{s}=8.45{}^{\circ }\pm 2.75{}^{\circ }$, the average absolute difference in tangent angles between points along the centerlines. We also find a correlation between these shape scores and *R*^2^ of the fit to the tip angle trajectory θ_tip_(*t*) (Supplementary Fig. S6), suggesting that fitting the root model to θ_tip_(*t*) alone also provides a good estimate for the whole shape of the root.

### Small angle approximation provides an analogy with a damped harmonic oscillator

In order to gain further insight, we analyze the root model in the limit of a small angle approximation $\left|\theta_{tip}\left(t\right)-\theta_{g}\right|\ll 1$. This is the variable of the sine function in Equation 7, enabling us to linearize the equation such that $-\left(k^{\prime }+\gamma k\right)\;/\eta =\int_{0}^{\tau }k\;\left(\tau^{*}\right)d\tau^{*}+k+{\tilde{\theta }}_{0}$. Taking a derivative in τ yields


$$\begin{array}{l} k^{\prime \prime }+(\eta \;+\;\gamma )k^{\prime }+\eta k=0 \end{array}$$ (11)

This can be identified as the equation for a damped harmonic oscillator, and the analogy provides an intuitive understanding of the dynamics. We identify two normalized time scales: (i) the natural angular frequency $\omega_{0}^{2}=\eta =\beta L_{gz}/r$, associated with the natural oscillations with no damping, and (ii) the attenuation rate Γ = (η + γ), associated with damping due to friction. Damped oscillations, corresponding to roots overshooting the direction of gravity, occur when $\omega_{0}^{2}>\Gamma^{2}/4$ [i.e. $\gamma <\left(2\sqrt{\eta }-\eta \right)$]. The quality factor is defined as $Q=\omega_{0}/\Gamma =\sqrt{\eta }/\left(\eta +\gamma \right)$, and describes how quickly oscillations die out. The quality factor of the observed root dynamics results in the low value of *Q* = 0.44 ± 0.16. We note that the time scale of the oscillations related to the natural angular frequency, $T_{\omega }=\frac{2\pi }{\omega_{0}}\frac{1}{{\dot{\varepsilon }}_{0}}=\frac{2\pi }{{\dot{\varepsilon }}_{0}}\sqrt{r/\beta L_{gz}}$, is proportional to the related timescale *T*_f_ estimated in Bastien *et al.* (2014) for the non-apical ACE model; the time required for an element aligned with the direction of gravity to leave the growth zone and become fixed. We can identify the source of the oscillations as the term η*k* in Equation 11, which can be traced back to the integral over the curvature exiting the growth zone in Equation 4. We note that neglecting the corresponding integral in Equation 6 recovers the AC model with no growth. Indeed, the tip angle in the apical AC model does not present oscillations and decays exponentially to its steady-state angle with an attenuation rate equivalent to η + γ (Fig. 3), showing that it is the damping term in Equation 11 which is responsible for the controlled turn towards gravity. The destabilizing source of oscillations therefore corresponds to *passive orientation drift* (Bastien *et al.*, 2014), where the tip angle of a growing organ with a fixed curvature increases, even with no differential growth. This drift leads to an overshoot of the direction of gravity, as clearly seen in Fig. 3A for low values of γ.

We note that the effective gravitropic sensitivity η = β*L*_gz_/*r* has opposite contributions to the shape dynamics: driving the root to align with gravity via the damping term, while also destabilizing the turn due to passive orientation drift, as it appears in both the natural angular frequency ω_0_ and the damping coefficient Γ. In contrast, the proprioceptive sensitivity γ appears only in the damping term, increasing posture control during the turn. Furthermore, following the theory of damped oscillations, the minimal amount of oscillations, which would lead to the fastest turn towards gravity, occurs in the case of critical damping, where $\gamma_{crit}=2\sqrt{\eta }-\eta$. We find $\langle \gamma_{crit}\rangle =0.9\pm 0.1$, which agrees with the estimated 〈
γ〉
=1.6±1.0, showing that on average proprioception may improve the tropic turn efficiency.

### Conclusions

Here we present a robust mathematical description of the spatio-temporal dynamics of plant root gravitropic responses. The model is based on physiological differences between roots and shoots informed by experiments on *A. thaliana*, namely apical sensing and a finite growth zone resulting in linear growth.

We show that the root model captures the dynamics of the tip angle, as well as the full spatio-temporal dynamics of the root’s gravitropic response over time. Furthermore, we find an analytical solution for the model, allowing estimation of the gravitropic and proprioceptive sensitivities, and we find that proprioception is necessary in order to describe root responses. This first dynamical quantification of proprioception in plant roots captures the observed dynamics of mutants associated with a lower proprioceptive gain (Okamoto *et al.*, 2015). This finding is significant since proprioception is poorly understood in plants and even less so in roots (Bastien *et al.*, 2013; Monshausen and Haswell, 2013; Okamoto *et al.*, 2015; Harmer and Brooks, 2018).

As stated in the Introduction, a mathematical model informed by experiments provides a number of advantages for the scientific community, and a valuable tool for hypothesis testing. Indeed, mathematical models are amenable to the use of mathematical analysis, as well as allowing analogies to entirely different systems with a similar mathematical description. For example, we found that in the limit of a small angle approximation, the root model is analogous to a damped harmonic oscillator (a system whose dynamics are very well understood), providing intuition for the observed dynamics. This analogy allows characterization of the decay rate of oscillations through the quality factor, as well as identification of the destabilizing source of oscillations as *passive orientation drift* (Bastien *et al.*, 2014), which leads to an overshoot of the direction of gravity. In turn, this overshoot is mitigated by proprioception. We find that the measured value for proprioception agrees with the value corresponding to critical damping of damped oscillators, exhibiting the minimal number of oscillations, suggesting the requirement of proprioception for efficient posture control in roots.

The model discussed here focuses on gravitropism, however, as discussed elsewhere (Porat *et al.*, 2020), it can be generalized to take into account other types of tropisms, such as hydrotropism and thigmotropism, as well as mechanical properties (Chelakkot and Mahadevan, 2017; Goriely, 2017; Sipos and Várkonyi, 2022; Zhang *et al.*, 2022). Taken together, this model provides a predictive tool which will allow the community studying plant tropisms to test hypotheses *in silico*, motivating future experiments—in line with recent advancements in quantitative plant biology (Autran *et al.*, 2021).

## Supplementary data

The following supplementary data are available at *JXB* online.

Table S1. Table of variables.

Fig. S1. Estimation of the growth zone length.

Fig. S2. Fitting tip angle trajectories to different models.

Fig. S3. Tip angle dynamics of a root with a triangular growth profile.

Fig. S4. Non-uniformity of curvature within the growth zone.

Fig. S5. Measure of the non-uniformity of curvature within the growth zone.

Fig. S6. Shape estimation quantification.

Video S1. Gravitropic turns given by the root model solutions with and without proprioception.

Video S2. Qualitative comparison of the full spatio-temporal dynamics.

erad383_suppl_Supplementary_Material

erad383_suppl_Supplementary_Video_S1

erad383_suppl_Supplementary_Video_S2

erad383_suppl_Supplementary_File

## Data Availability

The experimental angular trajectories data that support the findings of this study, and an example of a fitting algorithm to the root model, are openly available in Zenodo at https://doi.org/10.5281/zenodo.8189360; (Porat *et al*., 2023).
