# Supplementary material for: A quantitative model for spatio-temporal dynamics of root gravitropism
Source: J Exp Bot. 2023 Oct 23;75(2):620–30. doi: 10.1093/jxb/erad383 (PMC10773994; doi:10.1093/jxb/erad383)
Supplement: erad383_suppl_Supplementary_Material [file erad383_suppl_supplementary_material.pdf]

# – Supplementary Data –
# A quantitative model for spatio-temporal dynamics of root gravitropism

Amir Porat[a],Mathieu Rivière[b],Yasmine Meroz[b]

[a]School of Physics and Astronomy, Tel Aviv University, Tel Aviv 6997801, Israel
[b]School of Plant Sciences and Food Security, Tel Aviv University, Tel Aviv 6997801, Israel

## Contents

**Table S1. Table of variables** **2**

**Figure S1. Estimation of the growth zone length** **3**

**Figure S2. Fitting tip angle trajectories to different models** **4**

**Figure S3. Tip angle dynamics of a root with a triangular growth profile** **6**

**Figure S4. Non-uniformity of curvature within the growth zone** **6**

**Figure S5. Measure of the non-uniformity of curvature within the growth zone** **7**

**Figure S6. Shape estimation quantification** **7**

**Video S1. Gravitropic turns given by the root model solutions with and without proprioception** **8**

**Video S2. Qualitative comparison of the full spatio-temproal dynamics** **8**

## Table S1. Table of variables

| Notation | Definition | Mathematical definition |
|---|---|---|
| $t$ | time | |
| $s$ | arc length | |
| $v(s,t)$ | axial growth velocity | |
| $v_g$ | tip growth velocity | $v(s=L(t),t)$ |
| $L(t)$ | total length | |
| $L_{\text{gz}}$ | growth zone length | |
| $L_0$ | initial length | |
| $L_{\text{mz}}(t)$ | mature zone length | $L(t)-L_{\text{gz}}$ |
| $r$ | root radius | |
| $\theta(s,t)$ | local angle | |
| $\theta_{\text{tip}}(t)$ | angle at the apex | $\theta(s=L(t),t)$ |
| $\theta^0_{\text{gz}}(t)$ | angle at the base of the growth zone | $\theta(s=L(t)-L_{\text{gz}},t)$ |
| $\theta_{\text{f}}$ | steady-state angle | $\langle\theta_{\text{tip}}(t)\rangle_{t\geq 20h}$ |
| $\theta_{\text{g}}$ | angle of the stimulation | |
| $\theta_0$ | initial angle of the root | |
| $\kappa(s,t)$ | curvature | $\partial\theta(s,t)/\partial s$ |
| $\kappa_{\text{gz}}(t)$ | curvature in the growth zone | $\langle\kappa(s,t)\rangle_{s\in GZ}$ |
| $\kappa_{\text{mz}}(s)$ | curvature in the mature zone | |
| $\kappa^{\text{f}}_{\text{gz}}$ | steady-state value of $\kappa_{\text{gz}}$ | $\langle\kappa_{\text{gz}}(t)\rangle_{t\geq\tau}$ |
| $\tau$ | time to reach $\theta_{\text{f}}\pm\sigma(\theta_{\text{f}})$ | |
| $\dot{\varepsilon}_0$ | elongation rate in growth zone | |
| $\beta$ | gravitropic sensitivity | |
| $\gamma$ | proprioceptive sensitivity | |
| k | non-dimensional curvature | $L_{\text{gz}}\kappa_{\text{gz}}(t)$ |
| $\tau$ | non-dimensional time | $\dot{\varepsilon}_0 t$ |
| $\eta$ | effective gravitropic sensitivity | $\beta L_{\text{gz}}/r$ |

## Figure S1. Estimation of the growth zone length

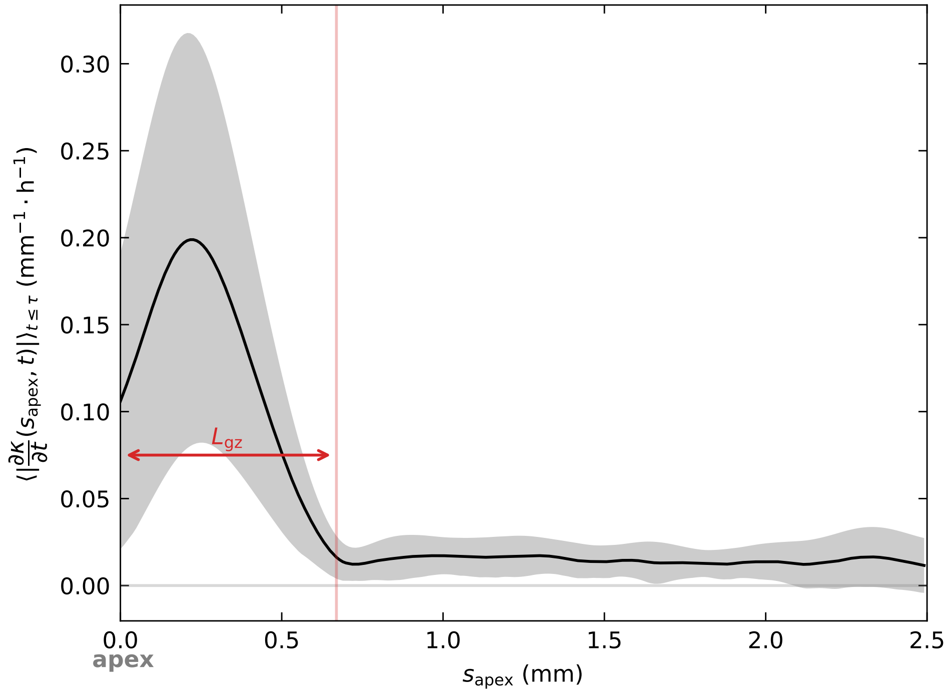


**Fig. S1. Variations of root curvature are circumscribed to a finite sub-apical region.** The absolute value of $\frac{\mathrm{d}\kappa}{\mathrm{d}t}$ is a function of time and the arc length defined from the root apex $s_{\mathrm{apex}}$. This quantity is averaged in time, for $t \leq \tau$, the typical timescale of the gravitropic response. The growth zone length $L_{\mathrm{gz}}$ is defined as the typical length over which curvature variations in time become negligible. Here $L_{\mathrm{gz}} = 0.67\mathrm{mm}$. Errors correspond to the standard deviation. This figure is built from the data in Fig. 3D.

## Figure S2. Fitting tip angle trajectories to different models

Here we find an expression for $\theta_{\text{tip}}(t)$ for the AC model and ACE model with exponential growth, in the case of apical sensing, solving the dynamical equations. These expressions are plotted in Fig.3B-C.

***AC model with apical sensing.*** The AC model with apical sensing follows (Bastien *et al.*, 2013)

$$\frac{d\kappa(t)}{dt} = \frac{\dot{\varepsilon}_0}{r}\left(-\beta\sin(\theta_{\text{tip}}(t)-\theta_{\text{g}}) - \gamma r\kappa(t)\right), \tag{S1}$$

for $s$ in the growth zone. We note that the model is written in this form in order to properly compare it to the root model. In the AC model growth is not considered explicitly, and the length of the organ is constant $L(t)=L_0$. In the case of apical sensing, an initially straight organ will develop constant curvature throughout the growth zone, and since $\theta_{\text{tip}}-\theta_0=\int_0^{L_0}\kappa ds$, we have

$$\theta_{\text{tip}}-\theta_0=\kappa L_0 \tag{S2}$$

Substituting Eq. S2 in Eq. S1, and integrating along $s$ following Bastien et al. (Bastien *et al.*, 2015) yields

$$\frac{1}{\dot{\varepsilon}_0}\frac{d\theta_{\text{tip}}}{dt} = -\frac{\beta L_0}{r}\sin(\theta_{\text{tip}}-\theta_{\text{g}}) - \gamma(\theta_{\text{tip}}-\theta_0). \tag{S3}$$

We take $\theta_{\text{g}}=\pi/2$, and the dynamics of $\theta_{\text{tip}}(t)$ can be found by

$$\dot{\varepsilon}_0 t = \int_{\theta_0}^{\theta_{\text{tip}}(t)} \frac{d\theta_{\text{tip}}}{\frac{\beta L_0}{r}\cos(\theta_{\text{tip}}) - \gamma(\theta_{\text{tip}}-\theta_0)} \tag{S4}$$

We note that the time diverges if $(\theta_{\text{tip}}-\theta_0)=\beta L_0\cos(\theta_{\text{tip}})/(r\gamma)$, giving an expression for the steady state tip angle. When $\gamma=0$, we obtain

$$\theta_{\text{tip}}(t) = \arcsin\left(\tanh\left(\frac{\beta L_0}{r}\dot{\varepsilon}t + c\right)\right) \tag{S5}$$

where $c=\text{artanh}(\sin(\theta_0))$. In the small angle approximation, Eq. S3 can be written as

$$\frac{1}{\dot{\varepsilon}_0}\frac{d\theta_{\text{tip}}}{dt} = -\left(\frac{\beta L_0}{r}+\gamma\right)\theta_{\text{tip}} + \frac{\beta L_0}{r}\theta_{\text{g}} + \gamma\theta_0 \tag{S6}$$

Such that the damping rate of the dynamics is $\beta L_0/r+\gamma$, similar to the non-dimensional rate $\eta+\gamma$ found in the main text for the root model. Lastly, in order to fit the tip angle trajectories to the dynamics of the AC model, we use a non-dimensional version of Eq. S3 by denoting $\tau=\dot{\varepsilon}_0 t$, $\frac{d\theta_{\text{tip}}}{d\tau}\equiv\theta'_{\text{tip}}$ and $\eta=\beta L_0/r$

$$\theta'_{\text{tip}} = -\eta\sin(\theta_{\text{tip}}-\theta_{\text{g}}) - \gamma(\theta_{\text{tip}}-\theta_0) \tag{S7}$$

Rearranging Eq. S8, taking arcsin on both sides and then taking another derivative of $\tau$ yields

$$-\frac{(\theta''_{\text{tip}}+\gamma\theta'_{\text{tip}})}{\sqrt{\eta^2-(\theta'_{\text{tip}}+\gamma(\theta_{\text{tip}}-\theta_0))^2}} = \theta'_{\text{tip}} \tag{S8}$$

We then fitted the tip angle trajectories to Eq. S8 as described in the methods for the root model. Note the similarity between Eq. S8 and Eq.8. We plot $\theta_{\text{tip}}$ for different values of $\gamma$ in In Fig.3B, and compare between the fitted values of $\gamma$ and $\beta$ of the AC model and the root model in Fig. S2.

***ACE model with exponential growth and apical sensing.*** Assuming a constant growth rate along the organ, we have $\frac{dL(t)}{dt}=\dot{\varepsilon}_0 L(t)$, leading to exponential growth

$$L(t)=L_0 e^{\dot{\varepsilon}_0 t} \tag{S9}$$

As before, curvature is constant for apical sensing, and the tip angle follows

$$\theta_{\text{tip}}(t)-\theta_0 = L_0 e^{\dot{\varepsilon}_0 t}\kappa(t). \tag{S10}$$

Substituting this expression in the ACE model in Eq.2 gives

$$\frac{d\kappa}{dt} = -\frac{\dot{\varepsilon}_0}{r}\left(\beta\sin(\theta_0-\theta_{\text{g}}+L_0 e^{\dot{\varepsilon}_0 t}\kappa) + \gamma r\kappa\right). \tag{S11}$$

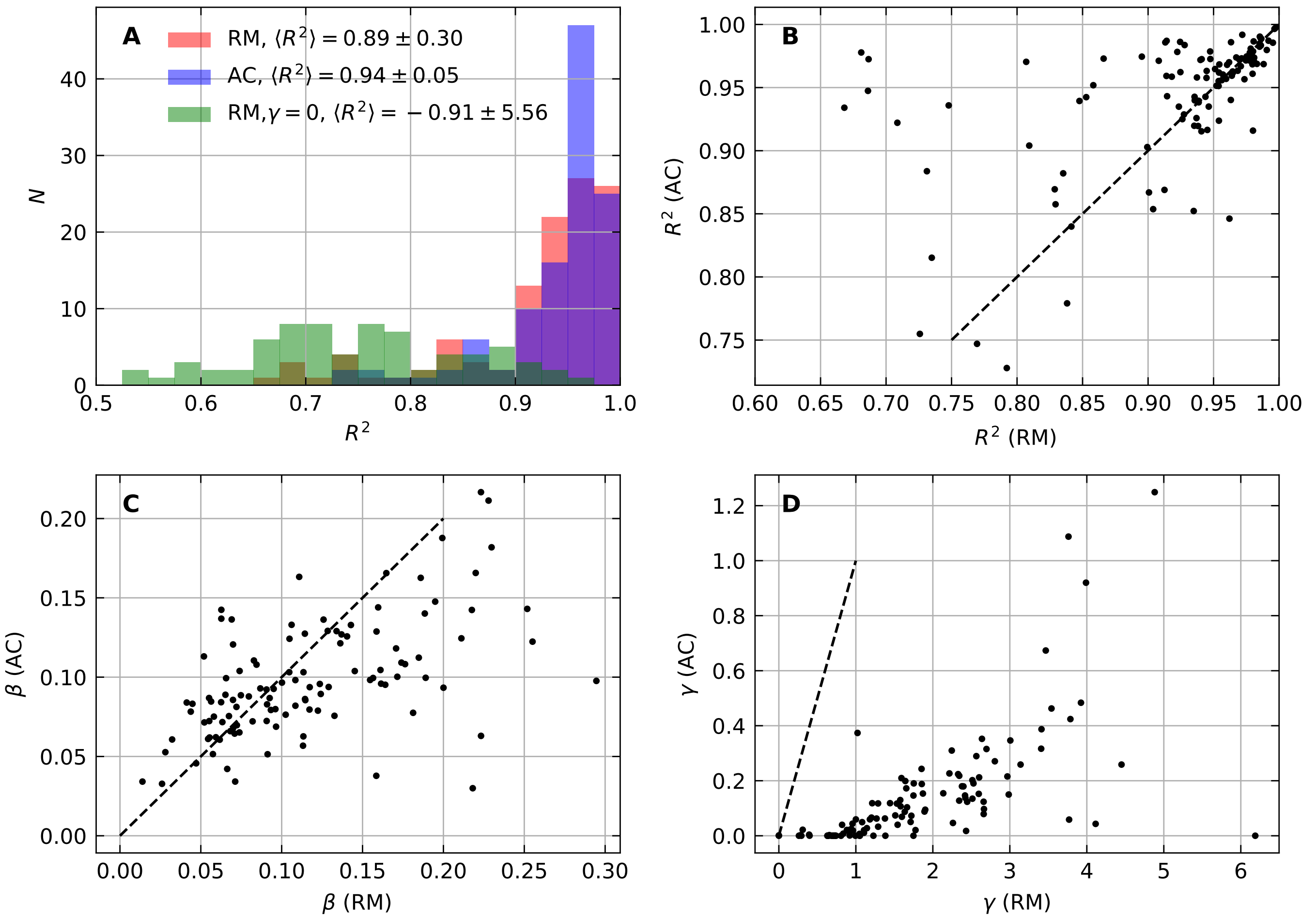


**Fig. S2. Comparison of parameters extracted from the root model and the AC model. (A)** Histograms of the coefficient of determination $R^2$ regarding the fitting of tip angle trajectories using the root model (RM), the root model without proprioception ($\gamma = 0$) and the AC model. **(B)** Comparison between $R^2$ estimated by the root model and the apical AC model. The dashed line is the identity function. **(C)** Comparison between $\beta$ estimated by the root model and the apical AC model. The dashed line is the identity function. **(D)** Comparison between $\gamma$ estimated by the root model and the apical AC model. The dashed line is the identity function.

Rewriting this expression in a non-dimensional form, substituting $\mathrm{k} = \mathrm{L}_0\kappa$, $\tau = \dot{\varepsilon}_0 t$ and $\eta = \beta L_0/r$ as in the main text gives

$$\frac{d\mathrm{k}}{d\tau} = -\eta \sin\left(\theta_0 - \theta_\mathrm{g} + e^{\tau}\mathrm{k}\right) - \gamma\mathrm{k}. \tag{S12}$$

This is an ODE with an initial condition $\mathrm{k}(0) = 0$. We note that k never reaches a steady state, and the angle oscillates at all times as shown in (Bastien *et al.*, 2014). In Fig.3C we integrate it by assuming $\theta_0 - \theta_\mathrm{g} = -\pi/2$. Fitting of the experimental data to Eq. S12 did not converge using standard fitting methods as described in the methods section.

## Figure S3. Tip angle dynamics of a root with a triangular growth profile

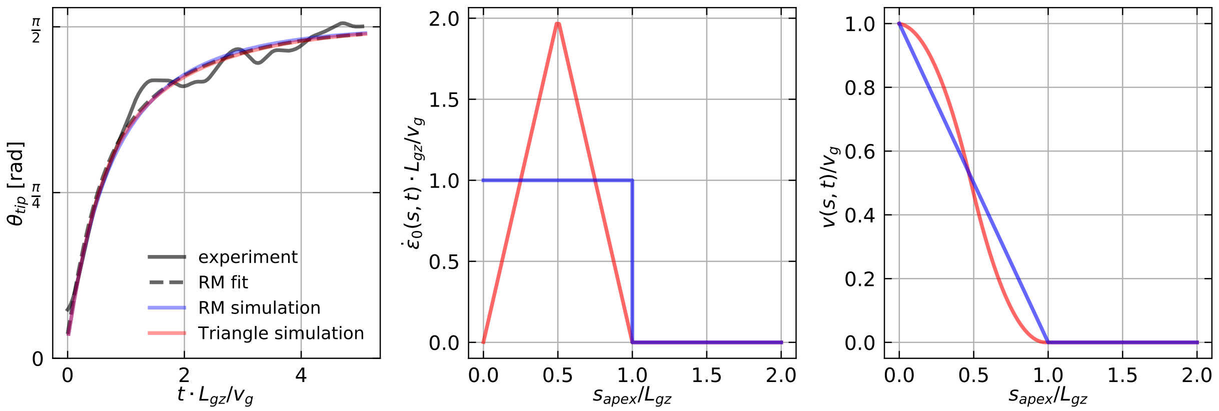


**Fig. S3. Tip angle dynamics of a root with a triangular growth profile.** Left: Comparison between the tip angle trajectories shown in the inset of Fig. 5A to two numerical simulations performed using the solver shown in (Porat *et al.*, 2020) and the same fitted parameters as in Fig. 5. One simulation has a uniform growth profile as in the root model, while the other simulation has a triangular growth rate profile, as can be estimated for our roots based on Fig. S1 and (Quiros *et al.*, 2022). Both simulations show negligible differences, verifying that a uniform growth rate is a good approximation, at least with respect to the tip angle dynamics. Center: the relative growth rate of both simulations, normalized by $v_g/L_{\mathrm{gz}}$, and non-trivial only in the growth zone, where $s_{\text{apex}}$ is the arc length defined from the root apex. Right: The growth velocity of both simulations, normalized by $v_g$, and non-trivial only in the growth zone.

## Figure S4. Non-uniformity of curvature within the growth zone

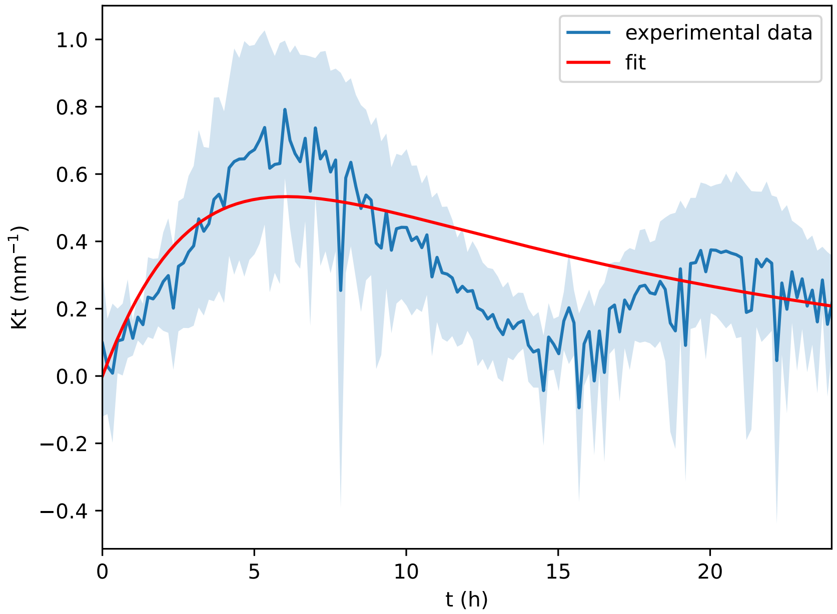


**Fig. S4.** Comparison of the experimental $\kappa_{\mathrm{gz}}$ to the fitted one. The dark blue line is the average measured curvature in the growth zone, and the light blue describes its standard deviation.

## Figure S5. Measure of the non-uniformity of curvature within the growth zone

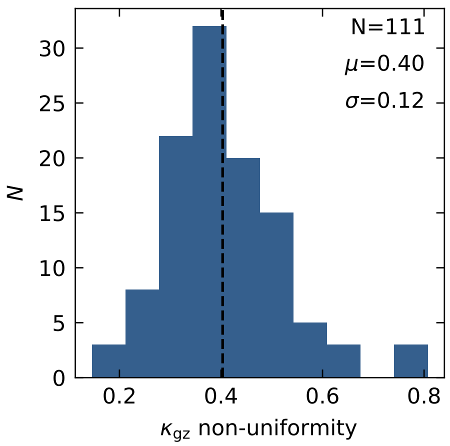


**Fig. S5. Measure of the non-uniformity of curvature within the growth zone.** The non-uniformity was defined as the noise to signal ratio $\sigma(\kappa_{\mathrm{gz}}(t))/\kappa_{\mathrm{gz}}(t)$ averaged over times $t$ such that $\kappa_{\mathrm{gz}}(t) \geq \kappa_{\mathrm{gz}}^{\mathrm{f}} + 2\sigma(\kappa_{\mathrm{gz}}^{\mathrm{f}})$, where $\kappa_{\mathrm{gz}}^{\mathrm{f}} = \langle \kappa_{\mathrm{gz}}(t) \rangle_{t \geq \tau}$

## Figure S6. Shape estimation quantification

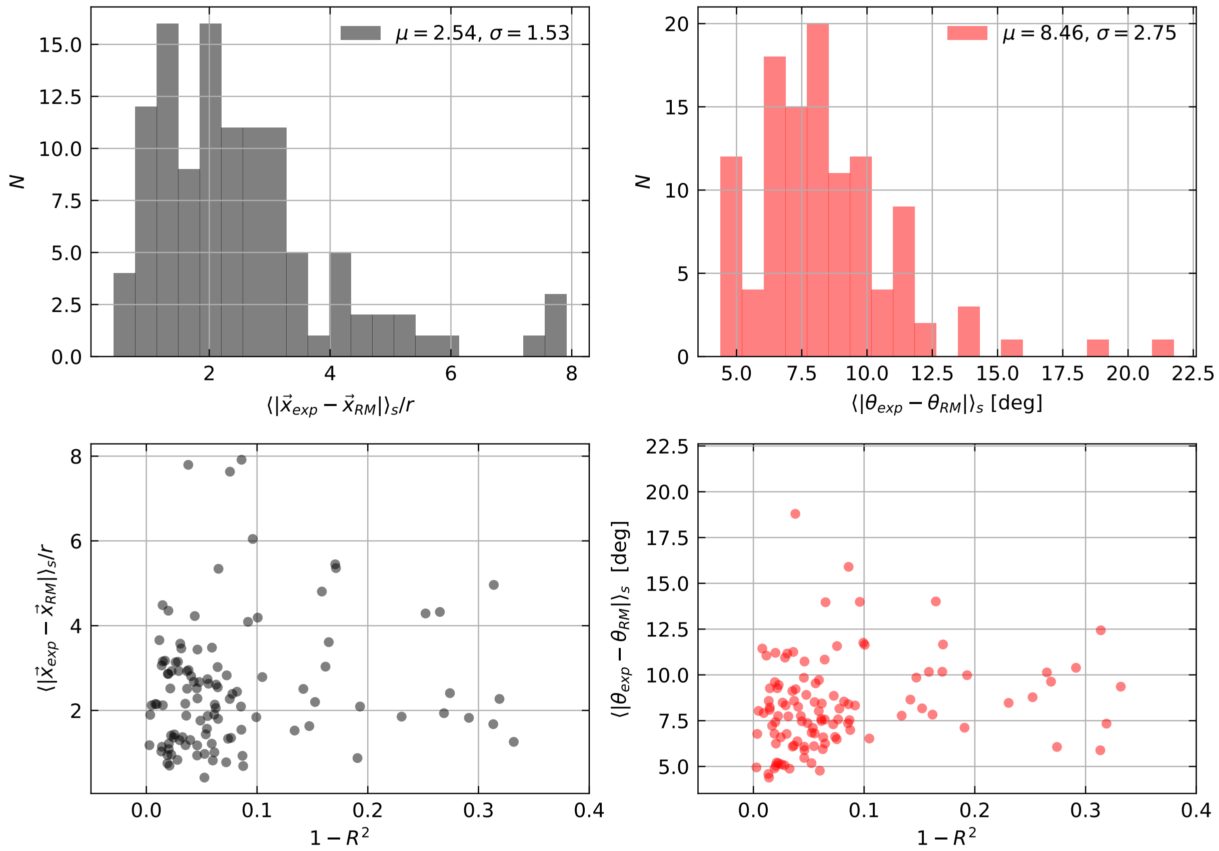


**Fig. S6. Errors of shape estimation by the root model.** Top left: Histogram of the average distance between points on each centerline with a similar arc-length from the initial base, normalized by the measured radius of the root, denoted by $\langle|\vec{x}_{\text{exp}} - \vec{x}_{RM}|\rangle_s / r$. Top right: Histogram of the average absolute value of the difference in tangent angles between points on each centerline with a similar arc-length from the initial base, denoted by $\langle|\theta_{\text{exp}} - \theta_{RM}|\rangle_s$. Bottom left: $\langle|\vec{x}_{\text{exp}} - \vec{x}_{RM}|\rangle_s / r$ plotted vs. $1 - R^2$, where $R^2$ is the coefficient of determination of the fit to the tip angle trajectories. The Pearson correlation coefficient is $0.32$. Bottom right: $\langle|\theta_{\text{exp}} - \theta_{RM}|\rangle_s$ plotted vs. $1 - R^2$, where $R^2$ is the coefficient of determination of the fit to the tip angle trajectories. The Pearson correlation coefficient is $0.50$.

## Video S1. Gravitropic turns given by the root model solutions with and without proprioception

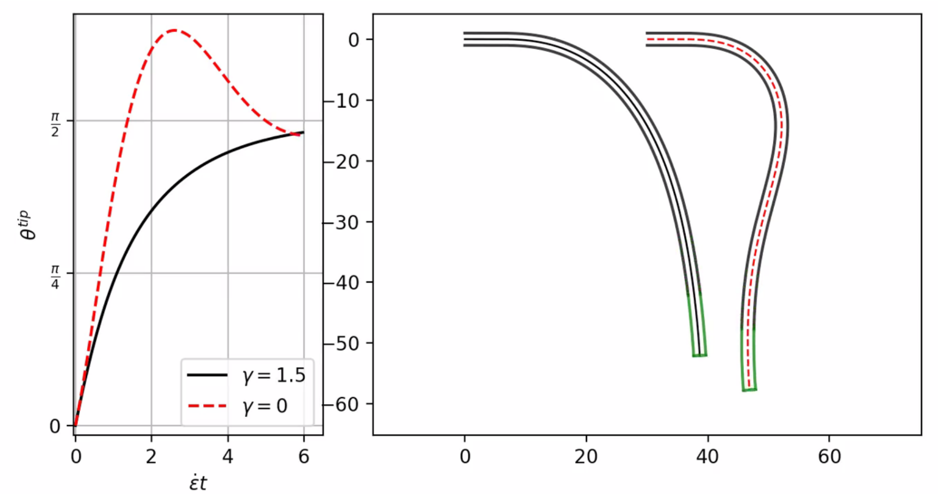


**Video S1.** Gravitropic turns given by the root model solutions with and without proprioception. Both roots have $\eta = 1$, $L_{\mathrm{gz}}/R = 10$ and $\beta = 0.1$ and different values of proprioceptive sensitivity $\gamma$. Left: Tip angle trajectories. Right: The corresponding root shapes, where the GZ is depicted in green while the MZ in black. Note that the root with $\gamma = 0$ overshoots the direction of gravity.

## Video S2. Qualitative comparison of the full spatio-temproal dynamics

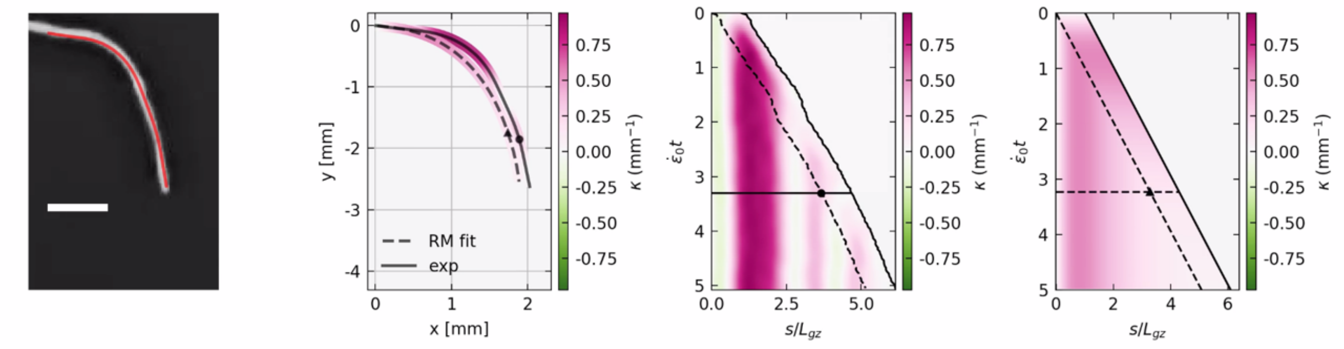


**Video S2.** Qualitative comparison of the full spatio-temproal dynamics. The three subfigures on the right are identical to Fig. 5A-C and are described there. Here, the growth zone is marked by a circle on the experimental centerline and by a triangle on the estimations from the root model. Left: Experimental image of the root overlaid by the estimation of its centerline in red (scalebar: 1 mm).

## Bibliography

**Bastien R**, **Bohr T**, **Moulia B**, **Douady S**. 2013. Unifying model of shoot gravitropism reveals proprioception as a central feature of posture control in plants. Proceedings of the National Academy of Sciences of the United States of America 110, 755–760.

**Bastien R**, **Douady S**, **Moulia B**. 2014. A unifying modeling of plant shoot gravitropism with an explicit account of the effects of growth. Frontiers in Plant Science 5.

**Bastien R**, **Douady S**, **Moulia B**. 2015. A Unified Model of Shoot Tropism in Plants: Photo-, Gravi- and Propio-ception. PLoS Comput Biol 11, e1004037.

**Porat A**, **Tedone F**, **Palladino M**, **Marcati P**, **Meroz Y**. 2020. A general 3d model for growth dynamics of sensory-growth systems: From plants to robotics. Frontiers in Robotics and AI 7, 89, doi: 10.3389/frobt.2020.00089.

**Quiros M**, **Bogeat-Triboulot MB**, **Couturier E**, **Kolb E**. 2022. Plant root growth against a mechanical obstacle: the early growth response of a maize root facing an axial resistance is consistent with the lockhart model. Journal of the Royal Society Interface 19, 20220266.
